# Rapid Preparation of Mesoporous Methylsilsesquioxane Aerogels by Microwave Heating Technology

**DOI:** 10.3390/molecules26071960

**Published:** 2021-03-31

**Authors:** Xingzhong Guo, Zixiao Li, Wei Lei, Ronghua Ding, Yun Zhang, Hui Yang

**Affiliations:** 1 State Key Laboratory of Silicon Materials, School of Materials Science and Engineering, Zhejiang University, Hangzhou 310027, China; 21826012@zju.edu.cn (Z.L.); yanghui@zju.edu.cn (H.Y.); 2 Pan Asia Microvent Tech (Jiangsu) Coporation & Zhejiang University Micro-Nano-Porous Materials Joint Research Development Center, Changzhou 213100, China; leiwei@microvent.com.cn (W.L.); dingronghua@microvent.com.cn (R.D.); zhangyun@microvent.com.cn (Y.Z.)

**Keywords:** MSQ aerogels, microwave drying, sol–gel

## Abstract

Microwave heating technology is known as an alternative to traditional gas and electric heating sources. In this work, mesoporous methylsilsesquioxane (MSQ) aerogels were prepared via a sol–gel process accompanied by microwave heating technology, and microwave heating was used in the gelation of sol and the drying of wet gels, respectively. The effects of hexadecyltrimethylammonium chloride (CTAC) as a surfactant and template, hydrochloric acid (HCl) as a catalyst, ethanol as a solvent, sodium hydroxide (NaOH) as a gelation agent, and microwave power on the pore structure of as-prepared MSQ aerogels were investigated in detail. Microwave heating at low power results in the acceleration of sol–gel transition and achieves the gelation within a few minutes. Appropriate amounts of chemical reagents and microwave heating at high power allow the preparation of mesoporous MSQ aerogels with a BET-specific surface area of 681.6 m^2^·g^−1^ and a mesopore size of 19 nm, and the resultant MSQ aerogel still has a BET specific surface area as high as 134 m^2^·g^−1^ after heat treatment at 600 °C for 2 h, showing high thermal stability. The MSQ aerogels/fibre composite possesses a low thermal conductivity of 0.039 W/(m·k)^−1^, displaying good thermal insulation. Microwave heating technology is a promising heating method for the preparation of other aerogels.

## 1. Introduction

Methylsilsesquioxane (MSQ) aerogel is a methyl hybrid silica aerogel prepared by sol–gel reaction containing methyl siloxane [1,2,3,4]. Compared with conventional silica aerogels, there are mutually exclusive methyl groups in MSQ aerogel framework that can improve its mechanical and intrinsic hydrophobic properties to some extent. MSQ aerogels have attracted widespread attention, especially in the fields of thermal insulation [5,6], adsorbent [7,8,9] and architecture [10,11,12].

In recent years, many studies have been reported about the optimization of synthesis process, pore structure, and properties of MSQ aerogels. Rao [13] reported the preparation of flexible MSQ aerogels by using trifunctional methyltrimethoxysilane (MTMS) as a precursor, oxalic acid as a catalyst, methanol as a solvent and ammonia as a gelation agent via supercritical drying. Nadargi [14] prepared the MSQ aerogels by using MTES as a precursor with a two-stage acid-base catalyzed sol–gel process followed by supercritical drying. Zhong [15] successfully prepared flexible MSQ aerogels with polydimethylsiloxane (PDMS) and MTMS as co-precursors via atmospheric pressure drying. Kanamori [16] reported the preparation of organic–inorganic composite MSQ aerogels by a two-step acid-base reaction. Kazuyoshi [17] introduced the preparation of MSQ aerogels by using the MTMS as a precursor, with and a cationic surfactant used to promote the mixing of the MTMS-derived species and the solvent mixture. In our previous works [18,19], flexible and elastic macroporous methylsilsesquioxane (MSQ) aerogels and translucent mesoporous MSQ aerogels with superhydrophobicity were successfully, prepared respectively. However, it should be noted that in most of the present reported MSQ aerogels, supercritical drying or ambient pressure drying was used to dry the wet gels. It is well known that supercritical drying needs special conditions such as high pressure and high temperature, which prevents aerogels from being used in industrial manufacturing and extended applications. Ambient pressure drying has become a popular research topic to replace supercritical drying for the preparation of aerogels, but low drying efficiency and poor structure retention limits its industrialization application. For these reasons, it is urgent to develop an effective heating method to achieve rapid drying of MSQ aerogels whilst retaining pore structure. 

In the present work, we use microwave heating technology to accelerate the sol-gel transition and fast drying of wet gel to prepare mesoporous MSQ aerogels. In the preparation process, the effects of catalyst (HCl), solvent (ethanol), surfactant (CTAC) and microwave drying power on the pore structure of MSQ aerogels are investigated in detail. The controllable preparation of mesoporous MSQ aerogels can be rapidly achieved by controlling starting compositions and using microwave heating technology. The MSQ aerogels/fibre composite is further prepared by the same starting compositions and drying method.

## 2. Materials and Methods 

### 2.1. Materials

Methyltrimethoxysilane (MTMS, Aladdin, Shanghai, China, 98%) and hydrochloric acid (HCl, Aladdin, Shanghai, China) were used as a precursor and a catalyst, respectively. Hexadecyltrimethylammonium chloride (CTAC, Aladdin, Shanghai, China, 97%) and sodium hydroxide (Aladdin, Shanghai, China, 97%) acted as a surfactant and a gelation agent, respectively. Ethanol (Aladdin, Shanghai, China, ≥99.5%), 2-propanol (Sinopharm Chemical Reagent Co., Ltd., Shanghai, China, ≥99.7%) and n-heptane (Sinopharm Chemical Reagent Co., Ltd., Shanghai, China, ≥98.5%) served as different kinds of solvent. All the chemical reagents were used as received.

### 2.2. Preparation of MSQ Aerogel and Aerogel/Fiber Composites

Preparation of MSQ aerogel: Table 1 shows the starting compositions and drying methods of all MSQ aerogel samples. A detailed preparation process was as follows. 0.24 g of CTAC, ethanol and HCl solution were uniformly mixed in a glass tube, and then 3.0 mL of MTMS was added with vigorous stirring in an ice-bath. After 30 min, the transparent solution (MSQ sol) was formed, and then NaOH solution was added to the solution to increase the pH value of the system. After stirring for 1 min, the as-prepared sol was gelated by microwave treatment at 350 W for 7 min. After gelation, the wet gels were sealed and kept at 60 °C for aging 24 h. All of the wet gels were solvent-exchanged with 2-propanol and n-heptane to remove water and CTAC twice, respectively, and then were dried by microwave at 500–700 W for 60 min to obtain the MSQ aerogel.

Preparation of MSQ aerogel/fiber composites: 24 g of CTAC, ethanol, and HCl solution were mixed in a glass tube, and then 300 mL of MTMS was added with vigorous stirring for 30 min in an ice-bath to form the transparent sol. NaOH solution was added in the sol to increase the pH value for the gelation of MSQ sol. After stirring for 1–2 min, the quartz fibers were completely immersed in the resultant sol, and the sol immersed fiber was gelated by microwave treatment at 350 W for 15 min. After gelation, the gel/fiber composite was sealed and kept at 60 °C for 24 h, and then was solvent-exchanged with 2-propanol and n-heptane to remove water and CTAC twice, respectively. Finally, the MSQ aerogel/fiber composite was prepared by microwave drying at 700 W for 75 min.

### 2.3. Characterization

The morphology of MSQ aerogels and composites was observed by a scanning electron microscope (SEM: Su8010, Hitachi, Tokyo, Japan). The Meso- and micropores of MSQ aerogels were characterized by an N_2_ adsorption–desorption apparatus (BET, ASAP2020HD88, Micromeritics Instruments Corporation, Norcross, GA, USA), and the samples were degassed at 200 °C under vacuum before adsorption–desorption measurement. The chemical compositions and thermal behaviour of MSQ aerogel were confirmed by Fourier-transform infrared spectroscopy (FT-IR, Nicolet 6700, ThermoFisher Scientific, Waltham, MA, USA), differential thermal analysis (DTA, Q200, TA, New Castle, DE, USA), and thermogravimetry (TG, TA-Q500, TA, New Castle, DE, USA). The thermal conductivity was obtained from a Hot Disk TPS 2500S.

## 3. Results and Discussion

### 3.1. Preparation Mechanism of Mesoporous MSQ Aerogels by Microwave Drying

**Figure 1:**
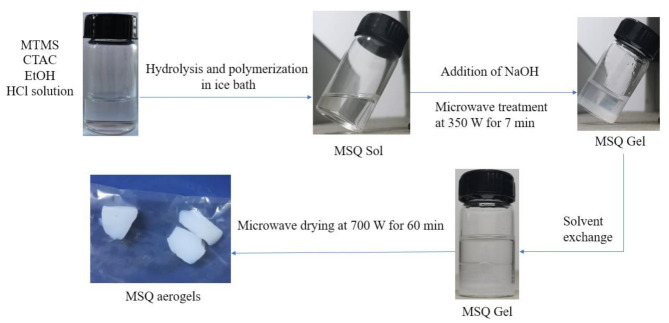
Schematic illustration of preparation process of methylsilsesquioxane (MSQ) aerogels by microwave drying.

Schematic illustration of rapid preparation of mesoporous MSQ aerogels by microwave drying was shown in Figure 1. In this acid-base system, CTAC is firstly dissolved in a mixture of ethanol and hydrochloric acid, and then the mixture is placed in ice bath until completely cooled. MTMS is added into the mixture and stirred for hydrolysis and polymerization. After that, NaOH solution is added to heighten the pH value of the system. In the meantime, the sol can be allowed to gelate after microwave treatment for a short time. After solvent replacement with 2-propanol and n-heptane and microwave drying, mesoporous MSQ aerogel can be obtained.

Microwave heating technology is a kind of heating method which uses electromagnetic wave as a heating source and the dried material itself as a heat generator. Compared with traditional heating methods through thermal radiation, microwave heating technology uniformly produces the heat by dielectric loss in electromagnetic field. Therefore, in this sol–gel process, the microwave heating technology can be used to not only accelerate the sol–gel transition but also achieve the fast drying of aerogels, which shortens the preparation procedure for aerogels. Microwave heating technology should be considered as an efficient gelation and drying technology for aerogels.

### 3.2. Optimization of Synthesis Process of MSQAerogels

#### 3.2.1. Effect of CTAC on Pore Structure of MSQ Aerogels

**Figure 2:**
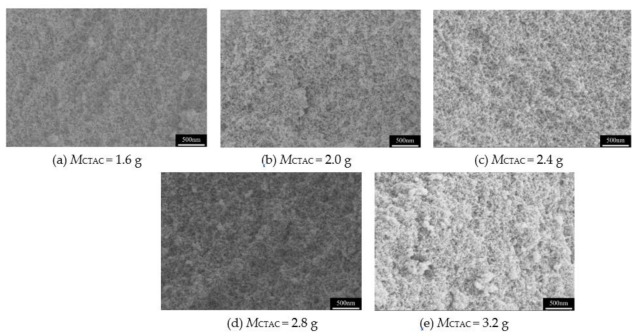
SEM images of MSQ aerogels prepared by 700 W microwave drying via sol-gel process with different CTAC contents. (**a**) 1.6 g, (**b**) 2.0 g, (**c**) 2.4 g, (**d**) 2.8 g, (**e**) 3.2 g.

Surfactant is known to have a beneficial effect on the formation of network frame, pore structure and particle size of aerogels. In this system, the CTAC is used as the surfactant for preparation of MSQ aerogels, and the effect of CTAC content on the pore structure of MQS aerogels is investigated. Figure 2 shows the pore structure of MSQ aerogels prepared via sol–gel process with different CTAC contents (M_CTAC_). We observed that all the MSQ aerogels possess a mesopore structure with uniform three-dimensional networks. With the increase in CTAC content, the size of mesopores increases, and the typical mesopore structure constructed by uniform gel network and homogeneous nanoparticles can be obtained when the CTAC content reaches 2.4 g. When the amount of CTAC further increases to 2.8 or 3.2 g, the mesopores of MSQ aerogels become smaller with some granular aggregates, indicating the deterioration of pore structure. The excessive involvement of CTAC in network construction resulted in a reduction in the liquid phase and accelerated the agglomeration of nanoparticles. As a result, it is concluded that 2.4 g of CTAC is an appropriate additive content of surfactant for the rapid preparation of MSQ aerogels.

#### 3.2.2. Effect of Ethanol on Pore Structure of MSQ Aerogels 

**Figure 3:**
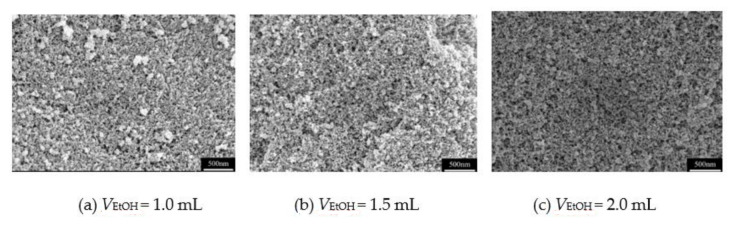
SEM images of MSQ aerogels prepared by 700 W microwave drying via sol-gel process with different volumes of ethanol: (**a**) 1.0 mL, (**b**) 1.5 mL, (**c**) 2.0 mL.

In previous works [15,16,17,18,19], methanol was often used as the solvent in the preparation of MSQ aerogels. However, methanol is a volatile chemical with certain toxicity, which will cause environmental pollution. Compared with methanol, ethanol has similar physical and chemical properties, and it is indeed less toxic. In this work, ethanol is used as the solvent for preparation of mesoporous MSQ aerogels, and the effect of ethanol volume on the pore structure of MSQ aerogel is analyzed. Figure 3 shows the pore structure of MSQ aerogels with different volumes of ethanol (V_EtOH_). It can be seen from the figures that as the content of ethanol increases gradually, the pore structure of MSQ aerogels has no obvious change, proving that the volume of ethanol has little relationship with the pore structure of MSQ aerogels. As a solvent, more ethanol will produce the tendency of phase separation, thus affecting the retention of pore structure in aerogels during drying. A small change in ethanol content does not affect the pore structure of MSQ aerogels obviously. In contrast, the MSQ aerogels with 1.5 mL ethanol show more uniform mesopore structure. This seems to conclude that 1.5 mL of ethanol is a suitable volume of solvent for rapid preparation of MSQ aerogels.

#### 3.2.3. Effect of Catalyst (HCl) on Pore Structure of MSQ Aerogels 

**Figure 4:**
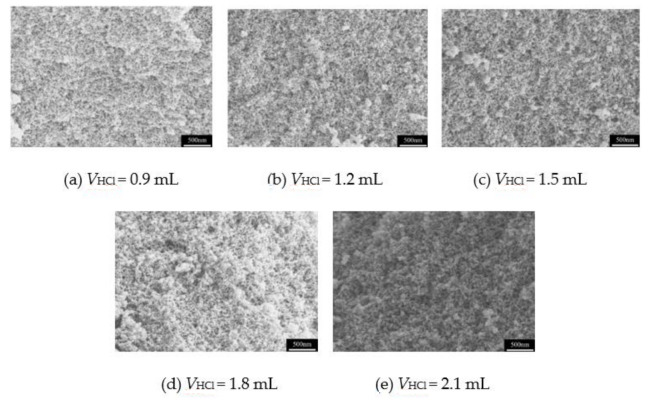
SEM images of MSQ aerogels prepared by 700 W microwave drying via sol-gel process with different volumes of HCl solution. (**a**) 0.9 mL, (**b**) 1.2 mL, (**c**) 1.5 mL, (**d**) 1.8 mL, (**e**) 2.1 mL.

In sol–gel reaction, the hydrochloric acid (HCl) is widely used as a catalyst for the hydrolysis of precursors. In this system, owing to the high exothermic reaction of MTMS hydrolysis, the reaction condition of an ice bath is essential to control the hydrolysis and polymerization, while at the same time, the hydrolysis of MTMS needs to be catalyzed by HCl. Here, the effect of additive volume of HCl on the pore structure of MSQ aerogels is inspected. Figure 4 shows the pore structure of MSQ aerogels with different volumes of HCl solution (*V*_HCl_). It is noted that with the increase in volume of HCl, the pore structure of MSQ aerogel does not change significantly. This may be due to the whole reaction being in the ice bath environment, the velocity of hydrolysis reaction is controlled very slowly. By comparison, the MSQ aerogels with 1.5 mL HCl solution show a more uniform mesopore structure. It can be concluded that 1.5 mL of HCl solution is an appropriate volume of catalyst for rapid preparation of MSQ aerogels.

#### 3.2.4. Effect of Sodium Hydroxide (NaOH) on Pore Structure of MSQ Aerogels 

**Figure 5:**
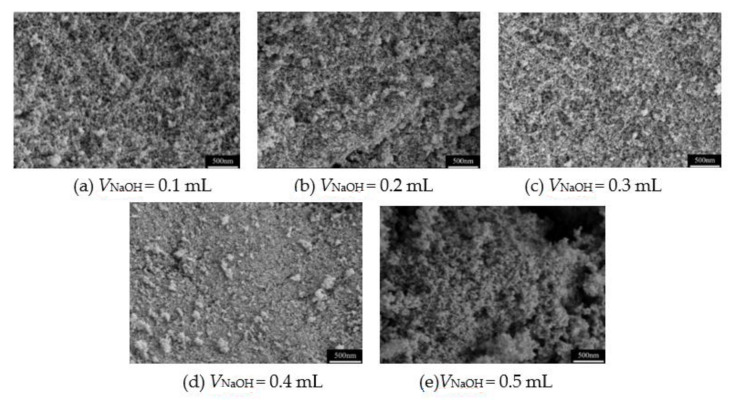
SEM images of MSQ aerogels prepared by 700 W microwave drying via sol-gel process with different volumes of 0.5 mol·L^−1^ NaOH solution. (**a**) 0.1 mL, (**b**) 0.2 mL, (**c**) 0.3 mL, (**d**) 0.4 mL, (**e**) 0.5 mL.

**Figure 6:**
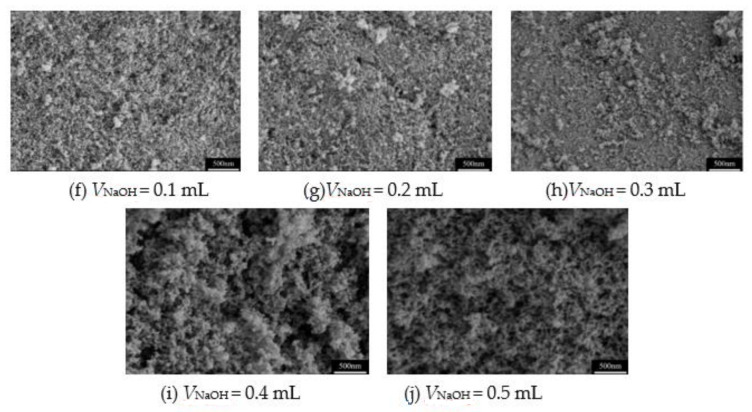
SEM images of MSQ aerogels prepared by 700 W microwave drying via sol-gel process with different volumes of 1.0 mol·L^−1^ NaOH solution. (**f**) 0.1 mL, (**g**) 0.2 mL, (**h**) 0.3 mL, (**i**) 0.4 mL, (**j**) 0.5 mL.

In our previous works [18,19], the propylene oxide (PO) was often used as the gelation agent in the preparation of MSQ aerogels. However, PO is also a volatile chemical with certain toxicity, which will cause environmental pollution. In this system, the frequently used NaOH solution is used as the gelation agent to accelerate the sol–gel transition, the effects of the volume and concentration of NaOH solution on the pore structure of MSQ aerogels are surveyed. Figure 5 and Figure 6 show the pore structure of MSQ aerogels with different volumes of 0.5 and 1.0 mol·L^−1^ NaOH solutions (V_NaOH_), respectively. For 0.5 or 1.0 mol·L^−1^ NaOH solution, with the increase in the volume of NaOH solution, the pore structure of both MSQ aerogels becomes more compact. In particular, when the volume of 0.5 mol·L^−1^ NaOH solution reaches 0.4 mL (0.3 mL for 1.0 mol·L^−1^ NaOH solution), the pore structure of MSQ aerogel is extremely dense. In contrast, MSQ aerogel has uniform pore structure with 0.2 mL of 0.5 mol·L^−1^ NaOH solution (0.1 mL of 1.0 mol·L^−1^ NaOH solution), indicating better volume and concentration of gelation agent for rapid preparation of MSQ aerogels. 

#### 3.2.5. Effects of Microwave Power and Drying Method on Pore Structure of MSQ Aerogels 

**Figure 7:**
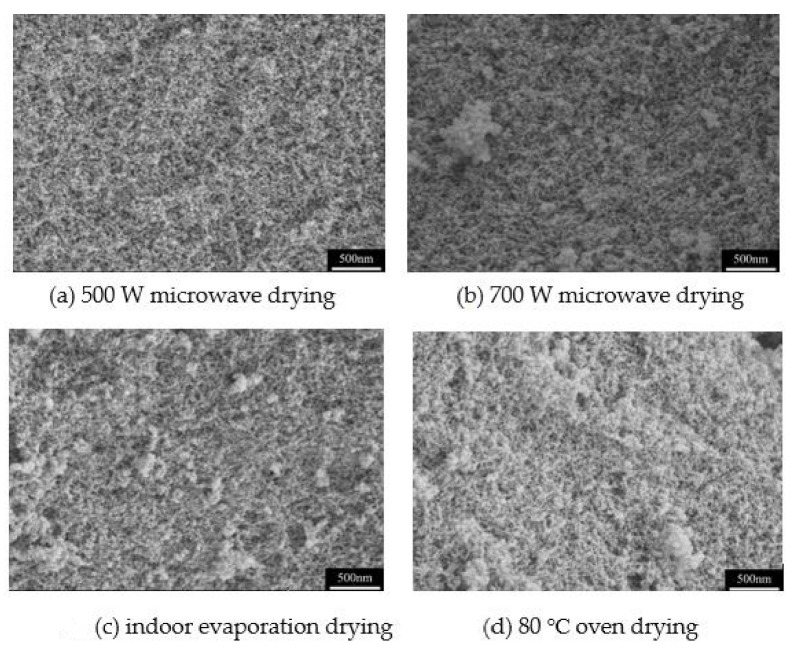
SEM images of MSQ aerogels prepared by microwave at different powers, indoor evaporation drying and oven drying. (**a**) 500 W microwave drying, (**b**) 700 W microwave drying, (**c**) indoor evaporation drying, (**d**) 80 °C oven drying.

Drying technology is one of the most important processes in aerogel preparation and determines the pore structure and production cost of aerogels. In this work, microwave heating technology is used to rapidly dry the wet gels, and the effect of microwave power on the pore structure of MSQ aerogels is researched. Additionally, the microwave drying is also compared with the indoor evaporation drying and oven drying. Figure 7 shows the pore structure of MSQ aerogels prepared by microwave at different powers, indoor evaporation drying and 80 °C oven drying, respectively. As can be seen from the figures, the MSQ aerogels prepared by microwave drying have more uniform pore structure than those dried by indoor evaporation drying or oven drying. Meanwhile, the power of microwave has little influence on the mesopores of MSQ aerogels. In contrast, the MSQ microwave dried at 500 W has more well-distributed pore structure than that at 700 W. It is certain that 500 W of microwave power is a suitable power of microwave drying for rapid preparation of MSQ aerogels.

**Figure 8:**
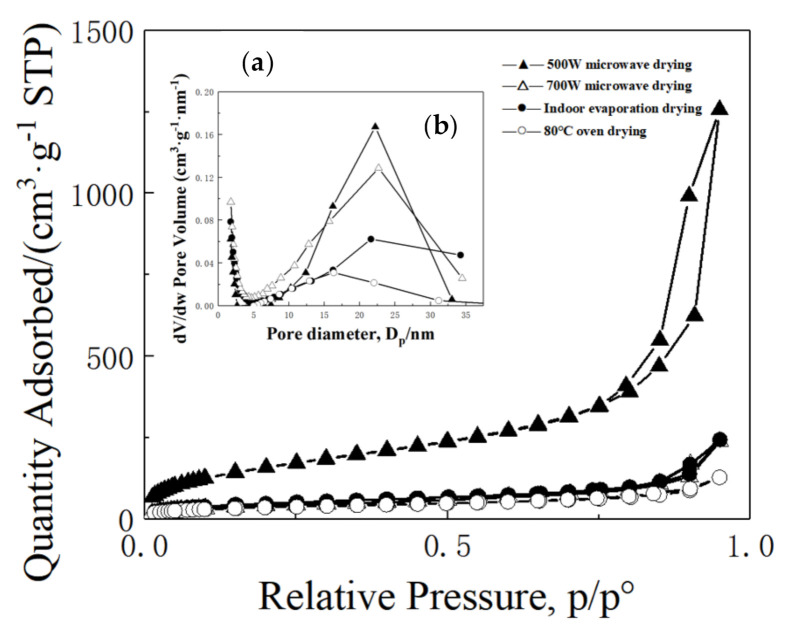
N_2_ adsorption–desorption isotherms (**a**) and BJH mesopore size distributions (**b**) of MSQ aerogels prepared by microwave at different powers, indoor evaporation drying and oven drying.

Figure 8 shows the N_2_ adsorption–desorption isotherms (a) and BJH mesopore size distributions (b) of different MSQ aerogels prepared by microwave at different powers, indoor evaporation drying and oven drying. It is clear that the MSQ aerogel prepared by microwave drying at 500 W presents a H_2_ adsorption–desorption hysteresis loop according to the IUPAC classification, confirming the existence of uniform and orderly mesopores. The calculated BET specific surface area and a mesopore size of MSQ aerogel by microwave drying at 500 W are 668 m^2^·g^−1^ and 19 nm, respectively. The BET specific surface area and a mesopore size of MSQ aerogel by microwave drying at 700 W are 557 m^2^·g^−1^ and 17 nm, respectively, which indicates that the micropores or mesopores decrease and the pore structure collapses at high microwave power. By comparison, the two MSQ aerogels prepared by indoor evaporation drying and oven drying show a dense microstructure and lower specific surface area. This confirms that microwave heating technology is an effective drying method for the rapid preparation of MSQ aerogels.

### 3.3. Thermal Stability and Pore Structures of Typical MSQ Aerogels

**Figure 9:**
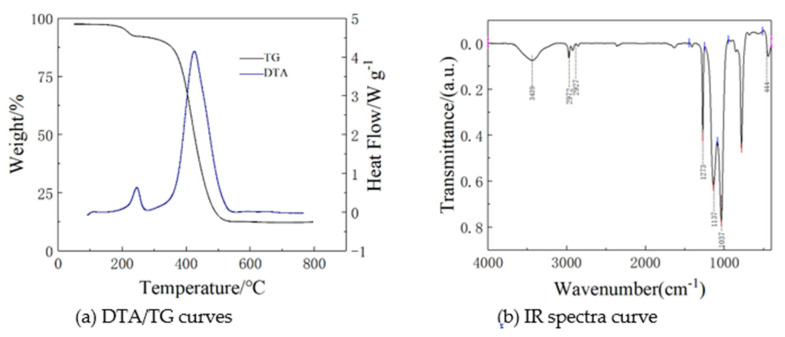
Differential thermal analysis (DTA)/thermogravimetry (TG) curves (**a**) and infrared (IR) specta curve (**b**) of a typical MSQ aerogel.

Figure 9 shows differential thermal analysis (DTA) and thermogravimetry (TG) curves of a typical MSQ aerogel. It is noted that when the temperature rises from 150 to 230 °C, there appears about 8% mass loss, which can be attributed to the evaporation of residual water. When the temperature further increases to 350 °C, the methyl groups of MSQ aerogel start to be oxygenated, which directly results in a particularly sharp peak in DTA curve and a nearly 80% mass loss. From infrared (IR) spectrum of the MSQ aerogels, the absorption peaks around 3439 and 1637 cm^−1^ are assigned as the stretching vibration and bending vibration of the O-H group of water, respectively. The absorption peaks around 2927 and 2972 cm^−1^ are attributed to the stretching vibration of the C-H group, and the absorption peaks around 1273 and 1137 cm^−1^ are due to the bending vibration of the C-H group, indicating the existence of a methyl group. The absorption peaks around 1137, 1037 and 444 cm^−1^ are ascribed to the stretching vibration of the O-Si-O group. The results confirm the successful preparation of pure MSQ aerogel.

**Figure 10:**
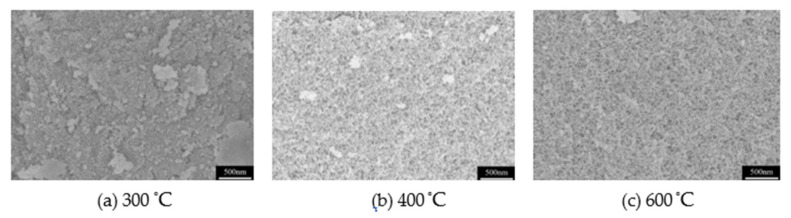
SEM images of MSQ aerogels prepared by 500 W microwave drying after heat-treated at different temperatures. (**a**) 300 °C, (**b**) 400 °C, (**c**) 600 °C.

The thermal stability of MSQ aerogel was investigated by heat-treating MSQ aerogel at different temperatures, and the pore structure change in heat-treated MSQ aerogels at 300–600 °C has been further studied. Figure 10 shows SEM images of typical MSQ aerogels after heat-treatment at different temperatures. It is seen that, with the increase in temperature, the pore structure of MSQ aerogels gradually shrinks and compacts. When the temperature reached 300 or 400 °C, the pore structure is not appreciably changed, but becomes very compact at 600 °C, which is the result of the exothermic reaction of methyl decomposition.

**Figure 11:**
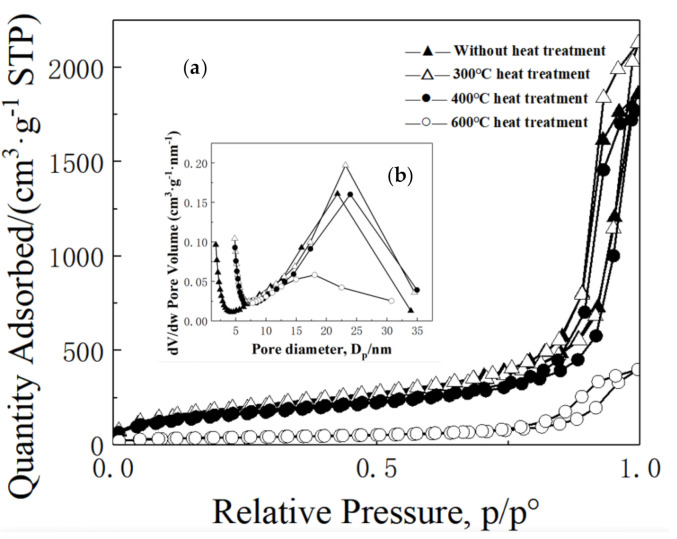
N_2_ adsorption–desorption isotherms (**a**) and BJH mesopore size distributions (**b**) of MSQ aerogels after heat treatment at different temperatures.

Figure 11 shows the N_2_ adsorption–desorption isotherms (a) and BJH mesopore size distributions (b) of typical MSQ aerogels heat-treated at varied temperatures. It is noted that the dried MSQ aerogel without heat-treatment presents a H2 adsorption-desorption hysteresis loop according to the IUPAC classification, confirming the existence of uniform and orderly mesopores. The calculated BET specific surface area and a mesopore size are 682 m^2^·g^−1^ and 19 nm, respectively. After heat-treatment at 300 and 400 °C, the isotherms and mesopore size distribution of MSQ aerogel have no obvious difference, while the overall shape of the adsorption–desorption hysteresis loop turns from H2 to H3, indicating the shrinking of mesopores and collapse of the pore structure after heat-treatment at 600 °C. The resultant MSQ aerogel after heat treatment at 600 °C for 2 h still has a specific surface area as high as 134 m^2^·g^−1^, showing high thermal stability.

### 3.4. Thermal Insulation Performance of MSQ Aerogel/Fiber Composites

**Figure 12:**
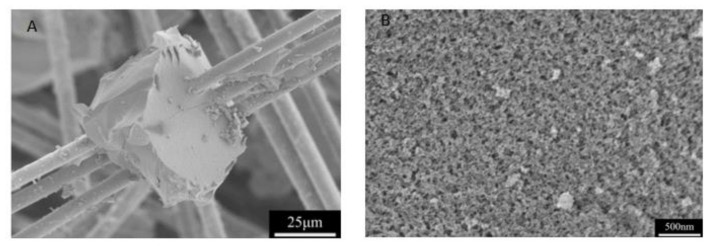
SEM images of the MSQ aerogel/fiber composites prepared by 700 W microwave drying. (**A**) combination of aerogel with fibre, (**B**) microstructure of aerogel in the composite.

Figure 12 shows the SEM image of MSQ aerogel/fiber composite. It is seen that some of the MSQ aerogels are attached on the surface of the fibers, and most of them fill in the network of the fibers or suspend on the fiber in the form of monoliths. Compared with the pure MSQ aerogel, the pore structure of MSQ aerogels in the composite become relatively denser, which possibly results from a collapse of mesopores or micropores during the microwave drying or a incomplete solvent exchange. The measured thermal conductivity of MSQ aerogel/fiber composite is 0.039 W/(m·K)^−1^, showing a high thermal insulation performance.

## 4. Conclusions

Microwave heating technology was used to accelerate the sol-gel transition and fast dry the wet gel to rapidly prepare methylsilsesquioxane (MSQ) aerogels with homogeneous mesoporous pores. In order to obtain a controllable and optimal formulation of MSQ aerogels, the influences of surfactant, catalyst, gelation agent, solvent, and microwave power on pore structure were thoroughly and carefully investigated. An appropriate proportion of MTMS:HCl:H_2_O:EtOH:CTAC:NaOH and microwave power of 500 W allow the preparation of MSQ aerogel with a specific surface area of 682 m^2^·g^−1^ and a mesopore size of 19 nm. The resultant MSQ aerogel displays high thermal stability, and a specific surface area still reaches 134 m^2^·g^−1^ after heat treatment at 600 °C. The MSQ aerogels/fibre composite has good thermal insulation with a thermal conductivity of 0.039 W/(m·k)^−1^. The microwave heating technology is promising for wider use in quick preparation of aerogels.

## Figures and Tables

**Table 1:** Starting compositions and drying methods of MSQ aerogels.

Sample	VMTMS /mL	VHCl /mL	VEtOH /mL	MCTAC /g	CNaOH /mol·L^−1^	VNaOH /mL	Microwave Drying Power/W
N1	3	1.5	1.5	1.6	0.5	0.5	700
N2	3	1.5	1.5	2.0	0.5	0.5	700
N3	3	1.5	1.5	2.4	0.5	0.5	700
N4	3	1.5	1.5	2.8	0.5	0.5	700
N5	3	1.5	1.5	3.2	0.5	0.5	700
N6	3	0.9	1.5	2.4	0.5	0.5	700
N7	3	1.2	1.5	2.4	0.5	0.5	700
N8	3	1.5	1.5	2.4	0.5	0.5	700
N9	3	1.8	1.5	2.4	0.5	0.5	700
N10	3	2.1	1.5	2.4	0.5	0.5	700
N11	3	1.5	1.0	2.4	0.5	0.5	700
N12	3	1.5	1.5	2.4	0.5	0.5	700
N13	3	1.5	2.0	2.4	0.5	0.5	700
N14	3	1.5	1.5	2.4	0.5	0.1	700
N15	3	1.5	1.5	2.4	0.5	0.2	700
N16	3	1.5	1.5	2.4	0.5	0.3	700
N17	3	1.5	1.5	2.4	0.5	0.4	700
N18	3	1.5	1.5	2.4	0.5	0.5	700
N19	3	1.5	1.5	2.4	1.0	0.1	700
N20	3	1.5	1.5	2.4	1.0	0.2	700
N21	3	1.5	1.5	2.4	1.0	0.3	700
N22	3	1.5	1.5	2.4	1.0	0.4	700
N23	3	1.5	1.5	2.4	1.0	0.5	700
N24	3	1.5	1.5	2.4	0.5	0.2	500
N25	3	1.5	1.5	2.4	0.5	0.2	700
N26	3	1.5	1.5	2.4	0.5	0.2	Indoor evaporation
N27	3	1.5	1.5	2.4	0.5	0.2	80 °C oven drying

V_MTMS_: volume of MTMS; V_HCl_: volume of HCl; V_EtOH_: volume of ethanol; M_CTAC_: mass of CTAC; V_NaOH_: volume of NaOH solution; C_NaOH_: concentration of NaOH solution.

## Data Availability

The data presented in this study are available on request from the corresponding author.

## Sample Availability

Samples of the compounds are not available from the authors.
